# Effects of Switching FSH Preparations on Sperm Parameters and Pregnancy: A Prospective Controlled Study

**DOI:** 10.3390/jcm13195666

**Published:** 2024-09-24

**Authors:** Rossella Cannarella, Claudia Leanza, Andrea Crafa, Antonio Aversa, Rosita A. Condorelli, Aldo E. Calogero, Sandro La Vignera

**Affiliations:** 1 Department of Clinical and Experimental Medicine, University of Catania, 95123 Catania, Italy; rossella.cannarella@phd.unict.it (R.C.); claudia.leanza.95@gmail.com (C.L.); rosita.condorelli@unict.it (R.A.C.); acaloger@unict.it (A.E.C.); sandrolavignera@unict.it (S.L.V.); 2 Glickman Urological & Kidney Institute, Cleveland Clinic Foundation, Cleveland, OH 44106, USA; 3 Department of Experimental and Clinical Medicine, University Magna Graecia of Catanzaro, 88100 Catanzaro, Italy; aversa@unicz.it

**Keywords:** FSH, hpFSH, rhFSH, pregnancy, therapeutic scheme, sperm concentration, total sperm count, switch

## Abstract

**Objective:** To study the effect of switching to a follicle-stimulating hormone (FSH) preparation other than that to which infertile male patients have not had an effective response. **Patients and methods:** Seventy-four normogonadotropinemic, non-obstructive, oligozoospermic patients who were poor responders to the administration of highly purified FSH (hpFSH) (Group 1 (n = 22) and Group 3 (n = 15)) or to recombinant human FSH (rhFSH) (Group 2 (n = 22) and Group 4 (n = 15)) were selected for this prospective study. After 3 months of washout from treatment with the first FSH preparation of choice, rhFSH was administered to patients in Groups 1 and 4 and hpFSH to those in Groups 2 and 3. Serum luteinizing hormone, FSH, total testosterone levels, conventional sperm parameters, testicular volume, and the number of pregnancies were evaluated at study entry and after the first and second treatment cycles. **Results:** Comparing treatment groups, the greatest improvement in sperm parameters was recorded in the groups of patients prescribed the switch in FSH preparation. Group 1 had the greatest benefit from therapy, with the highest pregnancy rate after the second treatment cycle. Indeed, eight couples achieved pregnancy (36.4%), compared to Groups 2 (n = 4; 18.2%), 3 (n = 1; 6.7%), and 4 (n = 2; 13.3%) (*p* = 0.04). **Conclusions:** The results of this study suggest that a therapeutic scheme involving the “switching” of the FSH preparation yields better results than a protocol using the same FSH preparation for six months. These findings, if confirmed by further studies, will help us better design a treatment strategy with FSH for infertile patients with oligozoospermia.

## 1. Introduction 

Infertility is defined as the inability to achieve pregnancy after 12 months or more of regular unprotected sexual intercourse [1]. According to the latest World Health Organization (WHO) report published in April 2023, one in six adults has experienced infertility in their lifespan. The estimated lifetime prevalence of infertility is 17.5%, with the highest prevalence in the WHO Western Pacific Region (23.2%) and the lowest in the WHO Eastern Mediterranean Region (10.7%) [2,3]. Male infertility affects approximately 50% of infertile couples, representing the only cause in about 30% of cases [4]. Despite an in-depth diagnostic process, the cause of male infertility is not known in a percentage that, depending on the completeness of the diagnostic workup, can reach up to 70% [5,6]. This condition is defined as idiopathic male infertility. 

The treatment of male infertility is challenging, especially in idiopathic cases, when no specific etiological factor is found during the diagnostic process. Non-hormonal treatment of male infertility, consisting of the use of nutraceuticals/antioxidants, is recommended in selected patients with idiopathic oligozoospermia and/or asthenozoospermia, and/or in those with signs of oxidative stress [7]. The rationale for hormonal treatment of male infertility stems from the notion that spermatogenesis requires the combined effect of adequate levels of follicle-stimulating hormone (FSH) and intratesticular testosterone (T), which in turn depends on luteinizing hormone (LH) stimulation [7,8]. In patients with hypogonadotropic hypogonadism, spermatogenesis is easily restored by the administration of gonadotropin hormone-releasing hormone or exogenous gonadotropins. The first is the most physiological therapy, but the use of gonadotropins represents a valid, less expensive, and more comfortable alternative. It consists of the administration of human chorionic gonadotropin (hCG) alone or in combination with FSH. Dosages and duration of treatment are still controversial, with some evidence suggesting slightly better outcomes from the combination of the two gonadotropins [8]. Current guidelines suggest the administration of hCG, selective estrogen receptor modulators, aromatase inhibitors, or a combination thereof for infertile patients with low serum T levels [9]. 

FSH is a therapeutic option available for male patients with idiopathic oligozoospermia or oligo-astheno-teratozoospermia (OAT) of non-obstructive origin and normal serum FSH levels [7,9,10]. FSH alone can be used in selected patients with oligozoospermia and/or asthenozoospermia, not due to obstructive seminal tract disease and serum of FSH levels < 8 IU/mL [7,11]. Indeed, infertile patients with serum FSH levels within the normal range may have a “clinical FSH deficiency” resulting from reduced activity of this hormone, which may depend on its glycosylation [11,12], FSHR polymorphisms, and other genetic variants [13]. Therefore, the rationale for prescribing FSH therapy in infertile patients with abnormal sperm parameters is to further stimulate spermatogenesis to induce an improvement in sperm production and/or quality [11,13]. International guidelines, however, indicate that the evidence on the use of FSH is still preliminary; it should be taken into consideration in trials but should not be routinely used in clinical practice [14].

Two different formulations of FSH are currently available: highly purified, urinary-derived FSH (hpFSH) and recombinant human FSH (rhFSH) [15]. Evidence in the literature does not suggest any difference between the two preparations when used for controlled ovarian hyper-stimulation in women undergoing assisted reproductive technique (ART) [16]. Similarly, a meta-analysis evaluating the effects of FSH dosage on conventional sperm parameters showed no difference in the efficacy between the two preparations [17]. A second meta-analysis evaluating pregnancy rate as the primary endpoint confirmed the efficacy of both FSH preparations when used to treat infertile patients [18]. 

Data from a clinical trial suggest that approximately half of patients with abnormal sperm parameters fail to respond to treatment with FSH [19]. Factors capable of predicting the response to FSH administration have therefore been sought to better select the patients to undergo treatment. Among these possible markers are serum FSH levels, *FSH receptor* (*FSHR*) and *FSH β-chain* (*FSHβ*) gene polymorphisms, and testicular histology. In short, patients with FSH levels within the normal range are believed to be more responsive to treatment, as high FSH levels (>10 IU/L) are indicative of primary spermatogenic failure, making FSH administration unnecessary [7,20]. Carriers of the polymorphism *FSHR* p. N680S (which makes the FSHR more capable of transducing the signal [21]) or the *FSHβ*-211 TT (associated with less efficient gene expression and lower FSH serum levels [22,23])) appear to show better response to FSH treatment [24,25]. Furthermore, patients with hypospermatogenesis associated with maturation arrest at the spermatid level appear to respond worse, unlike those with isolated hypospermatogenesis without maturation disturbances, who show a better response to FSH administration [19,26,27].

Regardless of the possible predictors of response to treatment (which, due to the low level of scientific evidence, find little use in clinical practice), one of the reasons for the poor response to therapy could be the lack of standardization of therapeutic protocols to be adopted. 

Various therapeutic schemes using FSH for infertile patients have been reported in the literature. Based on the weekly dosage, they can be divided into low doses (50 or 75 IU/L three times a week), intermediate doses (100 or 150 IU/L three times a week), and high doses (200 or 300 IU/L three times a week), usually for 3–4 months [17]. Although greater efficacy has been reported for higher doses [17], little is known about the doses and duration of treatment to achieve a better effect.

To date, no studies have investigated the effect of switching to an FSH preparation other than the one to which the patients showed a lack of response. Therefore, this study evaluated the effect of hpFSH in patients non-responsive to rhFSH or rhFSH in patients non-responsive to hpFSH. To accomplish this, we prospectively enrolled 74 patients with OAT without signs of obstruction and serum FSH levels < 8 mIU/mL who did not respond to hpFSH treatment (Group 1 (n = 22) and Group 3 (n = 15)) or to rhFSH (Group 2 (n = 22) and Group 4 (n = 15)), administered at a dose of 150 IU three times a week for three months. After 3 months of washout, we prescribed rhFSH to patients in Groups 1 and 4 and hpFSH to those in Groups 2 and 3 at a dose of 150 IU three times a week for another three months. The primary outcomes were conventional sperm parameters and pregnancy. At enrollment and the end of the first and second cycles of FSH therapy, luteinizing hormone (LH), FSH, and total testosterone (TT) and testicular volume (TV) were also measured and analyzed as secondary endpoints. 

## 2. Patients and Methods

### 2.1. Ethical Statement

This study was conducted at the Division of Endocrinology, Metabolic Diseases and Nutrition of the University-Teaching Hospital Policlinico “G. Rodolico-San Marco”, University of Catania (Catania, Italy). The protocol (n. 40/2022, approved on 12 January 2022) was approved by the internal Institutional Review Board. Informed consent was obtained from patients after a full explanation of the purpose and nature of all procedures used. The study has been conducted according to the principles expressed in the Declaration of Helsinki. 

### 2.2. Study Protocol 

The enrolled patients were treated with FSH for 3 months. Therefore, due to the poor response to the treatment—defined by the failure to achieve at least a doubling of the total sperm count compared to the values at enrollment [24] and/or no pregnancy—after 3 months of washout, they underwent another 3 months of FSH therapy. The washout was necessary, and it was not possible to carry out continuous therapy, as Italian legislation allows the free use of a specific formulation of FSH in patients with OAT only for 4 continuous months. 

In more detail, the patients were consecutively enrolled and randomly divided into four groups based on the type of FSH used and the sequence of use: Group 1 included patients treated with hpFSH (150 IU three times a week) for 3 months and, after a three-month washout, with rhFSH (150 IU three times a week) for another three months. Group 2, on the contrary, included patients who had started with rhFSH treatment (150 IU three times a week) and, after three months of washout, underwent hpFSH administration (150 IU three times a week). Group 3 and Group 4 included patients who started and continued (after the usual three months of washout) on the same FSH preparation (hpFSH and rhFSH, respectively). These latter two groups were considered control groups, as they did not undergo switching (Supplementary Figure S1). Assignment of patients to groups was carried out using a simple randomization method (www.randomizer.org, accessed on 16 February 2022). 

All parameters were assessed at baseline (T0), after the first three months of treatment (T1), and at the end of the second cycle of treatment. They included sperm concentration, total sperm count (TSC), sperm motility, sperm morphology, semen volume, number of patients achieving spontaneous pregnancy, TV, and serum levels of LH, FSH and TT. Age and body mass index (BMI) values were also collected. 

### 2.3. Patient Selection 

Patients with primary infertility, OAT, and normal serum FSH (<8 mIU/mL) and TT levels who did not respond to a 3-month course of treatment with hpFSH or rhFSH were enrolled in this study. Failure to respond to treatment was defined as when no pregnancy was achieved by 6 months after starting treatment. Patients were defined as oligozoospermic if they had a sperm concentration < 15 × 10^6^/mL or TSC < 39 × 10^6^/ejaculate, asthenozoospermic if they had progressive sperm motility < 32%, and teratozoospermic if they had spermatozoa with normal morphology < 4%, according to the WHO Manual, 5th edition [28]. Patients with central or primary hypogonadism, varicocele (all grades), urogenital infections (semen leukocyte concentration ≥ 1 × 10^6^/mL and/or positive semen culture), and forms of obstructive infertility were excluded, as well as those aged < 18 years with a history of being positive for or ongoing major comorbidities and/or organ failure (heart failure, kidney failure, liver failure, diabetes mellitus, and tumors) or with a history of or current alcohol abuse, drug use, or cigarette smoking. Patients whose female partner was ≥35 years old and those with serum anti-Müllerian hormone (AMH) levels below the lower limit of the reference range, tubal obstruction, or anovulation were excluded. Patients who had had previous pregnancies/live births were excluded. 

### 2.4. Hormonal Assessment

The blood tests were performed in the morning and while fasting, between 08:00 and 09:00, according to guidelines [29,30]. Serum levels of LH, FSH, TT, and AMH were assessed by using electrochemiluminescence. The reference ranges were 1.14–8.75 mIU/mL for LH, 0.95–11.95 mIU/mL for FSH, 250–877 ng/dL for TT, and 0.58–8.13 ng/mL for AMH. 

### 2.5. Testicular Volume Evaluation

TV was assessed by scrotal ultrasound using a high-frequency linear probe. After measuring the three diameters, the volume for each testis was calculated using the ellipsoid formula (length × width × thickness × 0.52). A volume of between 15 and 25 cm^3^ was considered normal, while a testis was considered hypotrophic if it had values < 10 cm^3^ [31]. The arithmetic mean TV value of the right and left testes was used for the statistical analysis.

### 2.6. Sperm Conventional Parameters

The semen analysis was performed in our Seminology laboratory. The collection of the seminal fluid was carried out by performing masturbation into a sterile container in a dedicated room after 2–7 days of ejaculatory abstinence. Two seminologists, who had received the same training and updated education according the 2010 WHO guidelines, analyzed the macroscopic and microscopic parameters immediately after liquefaction, which were evaluated according to the criteria of the 5th edition of the WHO manual for semen analysis [28]. Sperm concentration was assessed by placing an undiluted and well-mixed 10 μL semen sample in a clean Neubauer counting chamber maintained at 37 °C and at a magnification of 200× or 400×. Sperm motility was established by placing an undiluted semen sample inside a glass slide at 37 °C, covering it with a coverslip, and examining it at 200× magnification. By randomly scanning 200 spermatozoa, progressive and non-progressive sperm motility were evaluated. Total motility was obtained by the sum of the two. Morphology was assessed using the Papanicolaou staining procedure, while leucocyte concentration was assessed using the peroxidase assay.

### 2.7. Statistical Analysis

Data are shown as mean ± standard deviation (SD) for non-skewed variables, while non-normally distributed continuous variables are shown as median and interquartile range (IQR). The distribution of values was evaluated using the Shapiro–Wilk test. The treatment efficacy analysis in each group was carried out using one-way analysis of variance (ANOVA) or the Kruskal–Wallis test for normally or non-normally distributed variables, respectively, as appropriate. Comparative analysis between the four groups at each time point was performed using ANOVA or the Kruskal–Wallis test for normally or non-normally distributed variables, respectively. Analysis of differences in the number of pregnancies achieved in each group was performed using the Chi-squared test. Statistical analysis was carried out using MedCalc Software Ltd. (Ostend, Belgium), version 19.6-64-bit. A *p*-value of less than 0.05 was considered statistically significant.

## 3. Results

### 3.1. General Characteristics

Seventy-four patients with abnormal sperm parameters and normal FSH levels were enrolled in this study. Specifically, 67.6% (n = 50) had OAT, 31.1% (n = 23) had oligo-asthenozoospermia (OA), and 1.3% (n = 1) had asthenozoospermia. The mean age was 32.0 ± 7.9 years (range 18–51 years) and the mean BMI was 28.2 ± 5.9 Kg/m^2^ (range 18–41 Kg/m^2^). The baseline characteristics of the enrolled cohort are summarized in Supplementary Table S1. Patients were assigned to Group 1 (n = 22), Group 2 (n = 22), Group 3 (n = 15), or Group 4 (n = 15). All patients completed the study, and no dropouts were observed. No side effects were reported by any of the patients.

### 3.2. Intra-Group Analysis

The baseline parameters of the four groups are shown in Table 1.

**Figure 1:**
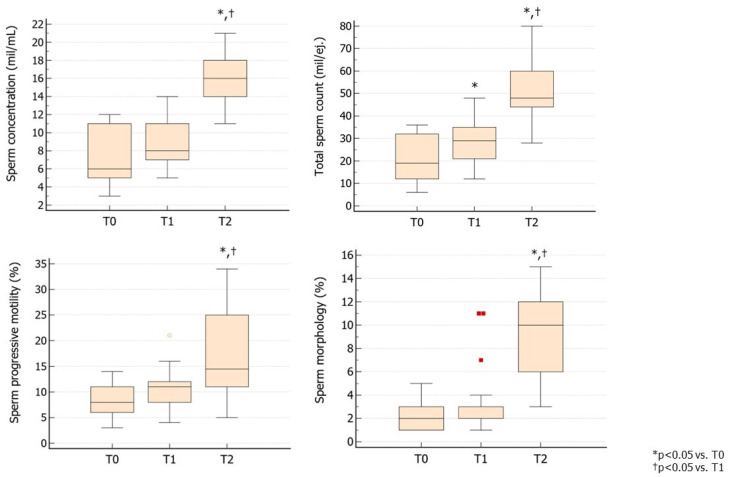
Conventional sperm parameters in Group 1. Group 1 consisted of patients treated with hpFSH followed by rhFSH at a dose of 150 IU three times a week for 3 months. The second cycle of therapy was prescribed after a 3-month washout from the first cycle (as per the provisions of the Italian Pharmacopeia) (Supplementary Figure S1). T0 shows parameters at baseline, T1 shows parameters after 3 months of hpFSH administration, andT2 shows parameters after 3 months of washout and a further 3 months of rhFSH administration. Abbreviations. FSH, follicle-stimulating hormone; hpFSH: highly purified FSH; rhFSH: recombinant human FSH.

In Group 1, treatment with hpFSH resulted in a significant increase in TSC, while sperm concentration, progressive motility, and morphology did not benefit from the therapy. Switching to rhFSH led to a significant improvement in sperm concentration, TSC, sperm motility, and morphology, all with significantly higher values compared to baseline or after hpFSH values (Figure 1). 

**Figure 2:**
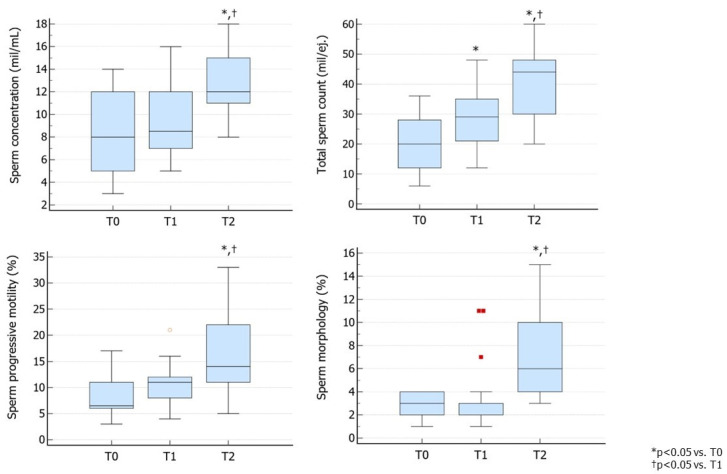
Conventional sperm parameters in Group 2. Group 2 consisted of patients treated with rhFSH followed by hpFSH at a dose of 150 IU three times a week for 3 months. The second cycle of therapy was prescribed after a 3-month washout from the first cycle (as per the provisions of the Italian Pharmacopeia) (Supplementary Figure S1). T0 shows parameters at baseline, T1 shows parameters after 3 months of rhFSH administration, and T2 shows parameters after 3 months of washout and a further 3 months of hpFSH administration. Abbreviations. FSH, follicle-stimulating hormone; hpFSH: highly purified FSH; rhFSH: recombinant human FSH.

Similarly, Group 2 did not benefit from treatment with rhFSH, since its administration resulted in a significant increase in TSC in the absence of improvement in sperm concentration, motility, and morphology. Following switching to hpFSH, a significant improvement in sperm concentration, TSC, progressive motility, and morphology was observed, indicating the efficacy of the switching scheme (Figure 2).

**Figure 3:**
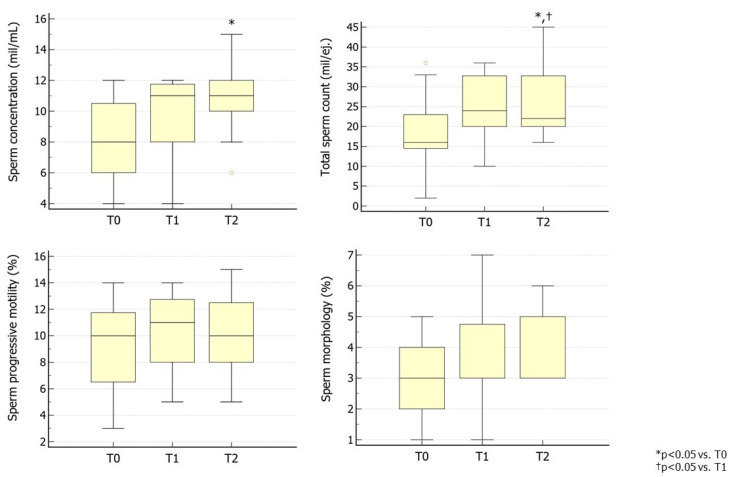
Conventional sperm parameters in Group 3. Group 3 consisted of patients treated with hpFSH followed by the same preparation at a dose of 150 IU three times a week for 3 months. The second cycle of therapy was prescribed after a 3-month washout from the first cycle (as per the provisions of the Italian Pharmacopeia) (Supplementary Figure S1). T0 shows parameters at baseline, T1 shows parameters after 3 months of hpFSH administration, and T2 shows parameters after 3 months of washout and a further 3 months of hpFSH administration. Abbreviations. FSH, follicle-stimulating hormone; hpFSH: highly purified FSH; rhFSH: recombinant human FSH.

In Group 3, the administration of hpFSH did not lead to any improvement in sperm parameters after 3 months. A second cycle of hpFSH, however, led to a significant improvement in sperm concentration and TSC without affecting progressive sperm motility and morphology (Figure 3). This may support some benefits of a prolonged hpFSH regimen on sperm parameters.

**Figure 4:**
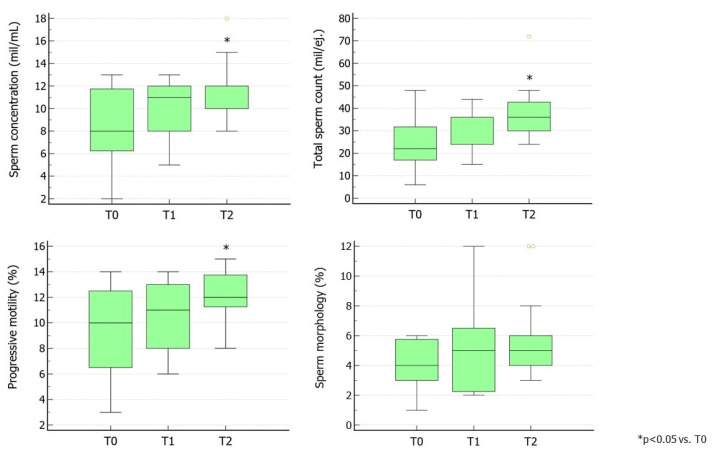
Conventional sperm parameters in Group 4. Group 4 consisted of patients treated with rhFSH followed by the same preparation at a dose of 150 IU three times a week for 3 months. The second cycle of therapy was prescribed after a 3-month washout from the first cycle (as per the provisions of the Italian Pharmacopeia) (Supplementary Figure S1). T0 shows parameters at baseline, T1 shows parameters after 3 months of rhFSH administration, and T2 shows parameters after 3 months of washout and a further 3 months of rhFSH administration. Abbreviations. FSH, follicle-stimulating hormone; hpFSH: highly purified FSH; rhFSH: recombinant human FSH.

Similarly, Group 4 showed no benefit from the first cycle of rhFSH therapy. After the second cycle, sperm concentration, TSC, and progressive motility improved significantly (Figure 4), again supporting the benefit of a prolonged rhFSH regimen on sperm parameters.

Group 1 showed significantly higher FSH, TT, and TV levels at T2 compared to their respective values at T0 and T1. Serum LH levels did not differ significantly. In Group 2, FSH and TV increased significantly at T2 compared to the values at T0 and T1. LH at T2 was significantly higher than the values at T0 but did not differ from the levels at T1. No difference in TT was found between levels at each time point. In Group 3, FSH and LH at T2 were higher than the values at T0 but did not differ from the levels at T1. TT and TV were not significantly different. Group 4 showed that the FSH, LH, TT, and TV values were significantly higher at T2 compared to T0. The LH and TT values at T1 were also significantly higher than those at T0 (Table 2).

### 3.3. Intergroup Analysis

None of the parameters of the various groups differed significantly from each other at baseline, making the groups comparable (Table 1). 

At T1, none of the sperm parameters were different between groups. FSH levels were higher in Group 4 than in Group 1. LH showed significantly higher levels in Groups 3 and 4 than in Groups 1 and 2. TT and VT did not differ between groups (Table 3). At T1, the number of pregnancies was zero in all groups, as per the inclusion criteria.

**Figure 5:**
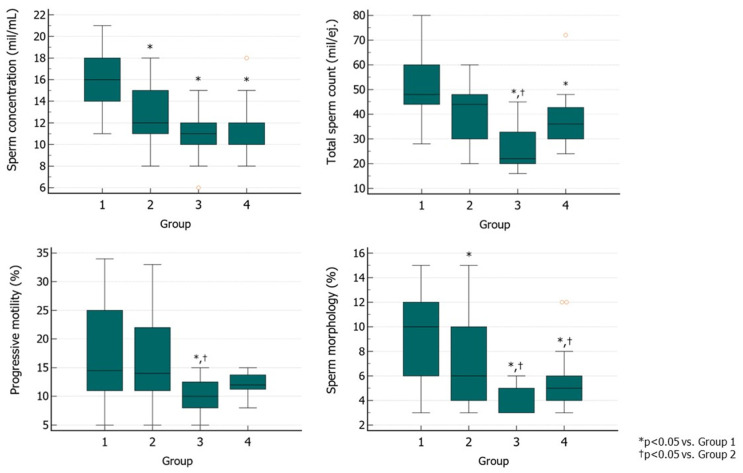
Sperm parameters at the end of the second treatment cycle (T2) with hpFSH followed by the administration of rhFSH (Group 1), rhFSH followed by the administration of hpFSH (Group 2), (hpFSH followed by the administration of the same FSH preparation (Group 3), and rhFSH followed by administration of the same FSH preparation (Group 4). The two different FSH preparations were administered at a dose of 150 IU three times a week for 3 months, and the second cycle of therapy was prescribed after a three-month washout from the first for all groups (as per the provisions of the Italian Pharmacopeia) (Supplementary Figure S1). Abbreviations. FSH, follicle-stimulating hormone; hpFSH: highly purified FSH; rhFSH: recombinant human FSH.

At T2, Group 1 benefited most from the therapy in terms of amelioration of conventional sperm parameters. The sperm concentration in Groups 2, 3, and 4 was significantly lower than in Group 1. TSC was significantly lower in Groups 3 and 4 than in Group 1. Group 3 had also significantly lower values than Group 2, while no differences were found between Groups 1 and 2. Group 3 showed a significantly lower progressive motility than Groups 1 and 2. Groups 3 and 4 had worse morphology than Groups 1 and 2, while Group 2 had lower morphology than Group 1 (Figure 5). 

Regarding hormone values, serum FSH and TT levels did not differ between groups. LH was significantly higher in Group 3 than in Group 1, while Group 4 showed significantly higher levels than Groups 1 and 2 (Supplementary Table S2). 

Group 1 reported the highest number of spontaneous pregnancies, with a total of eight couples (36.4%). Four pregnancies were registered in Group 2 (18.2%), one in Group 3 (6.7%), and two in Group 4 (13.3%) (*p* = 0.04).

## 4. Discussion 

In the present study, we evaluated the effectiveness, in terms of sperm parameters and pregnancies, of switching from one FSH preparation to the other available after three months of washout. The washout was mandatory due to the current regulation on FSH treatment for idiopathic male infertility in Italy, which does not allow the treatment to be prescribed continuously.

The four groups were comparable, as none of the patient parameters differed significantly from each other at baseline. After the first three months of treatment with hpFSH or rhFSH, respectively, TSC showed a significant increase in Groups 1 and 2, but not in Groups 3 and 4. This difference between the groups treated with the same FSH preparation and the same posology (Groups 1 and 3 treated with hpFSH 150 UI three times a week, Groups 2 and 4 treated with rhFSH 150 UI three times a week) could derive from inter-individual response variability. However, no pregnancies were recorded in any groups. 

After the second cycle of treatment, the two groups that underwent FSH preparation switch showed a significant improvement in sperm concentration, TSC, progressive sperm motility, and sperm morphology compared to baseline, while the groups that continued with the same preparation prescribed for the first cycle showed an improvement only in sperm concentration and TSC compared to baseline. These results support the effectiveness of a prolonged regimen with hpFSH or rhFSH, especially on sperm count. However, when all groups were compared at the end of the second cycle, the improvement in sperm parameters was more marked in the switched groups. More specifically, Group 1 showed the maximum benefit from FSH treatment, reporting a higher pregnancy rate after the second cycle of treatment, with a total of eight patients whose female partners had achieved pregnancy (36.4%), followed by Group 2 (4 pregnancies), Group 4 (2 pregnancies), and Group 3 (1 pregnancy). These results indicate the superiority of the “switching” scheme compared to the use of the same preparation for the six months of treatment. Furthermore, they suggest that the best therapeutic scheme involves administration of hpFSH for the first 3 months and rhFSH for the other 3 months, both at a dose of 150 IU three times a week.

The reasons for the results of the present study are not immediately clear. The molecular pharmacology of FSHR itself is not yet well understood [32]. Our findings led us to hypothesize that a sort of desensitization of the FSHR to the preparation used initially could be overcome either by prolonging the duration of exposure to FSH or through the administration of a different preparation, with greater efficacy in the latter case. The lack of knowledge of the mechanism of saturation and desensitization of the FSHR is also underlined by the existence of the so-called “suboptimal ovarian response”, a condition of unclear etiology that requires further investigation. The suboptimal ovarian response to FSH describes young patients without factors predictive of poor response to gonadotropins, for which an increase in FSH dosage is necessary to recruit a sufficient number of oocytes during controlled ovarian hyperstimulation in ART cycles [32,33]. To date, no study has evaluated the effect of an FSH switching protocol in infertile patients, so it is not known whether this condition can be ascribed to the habituation of the FSHR to the specific FSH preparation used.

Interestingly, a possible explanation for our results could be the different molecular structures of the FSH formulations administered to the patients enrolled in this study. As is already known, hormones regulate the expression of specific genes in endocrine-dependent cells, and this regulation can vary according to their molecular structure. FSH is constituted by a family of glycosylation variants, which differ in their oligosaccharide structure [34]. Different molecular structures have shown complementary and specific actions on follicle development [35,36]. Evidence in the literature based on the micro-array approach suggests that the expression of several genes in granulosa cells of women undergoing controlled ovarian hyperstimulation is affected by the type of FSH used (urinary or recombinant) [37,38]. The difference in gene expression may arise from the different molecular structure between urinary and recombinant FSH, which in turn differ in their carbohydrate structures [39,40]. The carbohydrate component is important in several functions, including regulation of metabolic clearance or receptor-binding activity, biological signal transduction, and modulation of hormone potency [39]. Therefore, changes in these components may have repercussions on all these functions and ultimately on the effect of FSH treatment. This may partly explain the results obtained in this study.

The presence of control groups and the lack of differences between groups at baseline represent the strengths of the study. A limitation is the need to discontinue FSH administration for three months, but we could not do otherwise considering the regulation of the Italian Pharmacopeia on this aspect. Another aspect that could influence our results is the different responses shown by the four groups after three months of therapy. Indeed, Groups 1 and 2, probably due to inter-individual variability, obtained better results after the first cycle of treatment and, therefore, started the second cycle of therapy obtaining better parameters compared to the control groups, which could have influenced the final results at the end of the other three months of treatment. However, the groups did not differ in terms of the number of pregnancies at three months. 

In conclusion, our study adds novel evidence that could be useful for the definition of a precise therapeutic scheme for the hormonal treatment of idiopathic male infertility. They suggest the superiority of the “switching” scheme compared to the use of the same preparation for six months of treatment and suggest as the best therapeutic scheme the administration of hpFSH for the first 3 months and of rhFSH for the other 3 months, both in a dose of 150 IU three times a week. Further studies are needed to confirm the results of the present study and to understand the molecular causes underlying our findings.

Capsule: Switching from one FSH preparation to which the infertile male patient showed a poor response profile to another proved effective on sperm parameters and spontaneous pregnancy, with the best efficacy obtained from the hpFSH-to-rhFSH scheme.

## Figures and Tables

**Table 1:** Baseline characteristics of patients included in each group. Group 1 was treated with hpFSH followed by rhFSH, Group 2 with rhFSH followed by hpFSH, Group 3 with hpFSH followed by the same preparation, and Group 4 with rhFSH followed by the same preparation. The two different FSH preparations were administered at a dose of 150 IU three times a week for 3 months, and the second cycle of therapy was prescribed after a 3-month washout from the first cycle for all groups (as per the provisions of the Italian Pharmacapeia) (Supplementary Figure S1).

	Group 1	Group 2	Group 3	Group 4	*p*-Value
Age (years)	32.7 ± 8.4	34.2 ± 9.0	29.5 ± 6.3	30.3 ± 6.0	0.25
BMI (kg/m^2^)	25.7 (23.0–34.0)	30.0 (20.0–33.0)	27.0 (23.3–33.8)	27.0 (23.0–33.8)	0.66
Testicular volume (mL)	11.1 ± 3.0	11.3 ± 2.5	9.9 ± 2.3	9.6 ± 3.3	0.2
LH (mUI/mL)	2.8 ± 0.9	2.8 ± 1.0	3.1 ± 0.8	3.3 ± /0.1	0.2
FSH (mUI/mL)	3.0 (2.2–3.8)	3.0 (2.3–3.8)	2.8 (2.2–3.3)	3.3 (2.4–4.2)	0.66
Total testosterone (ng/dL)	482.5 (445.0–555.0)	549.5 (456.0–613.0)	513.0 (452.0–565.0)	469.0 (443.5–562.0)	0.28
Sperm concentration (mil/mL)	6.0 (5.0–11.0)	8.0 (5.0–12.0)	8.0 (6.0–10.5)	8.0 (6.3–11.8)	0.68
Total sperm count (mil/ejaculate)	19.0 (12.0–32.0)	20.0 (12.0–28.0)	16.0 (14.5–23.0)	22.0 (17.0–31.8)	0.50
Progressive motility (%)	8.7 ± 3.2	8.0 ± 3.5	9.1 ± 3.5	9.3 ± 3.5	0.64
Morphology (%)	2.0 (1.0–3.0)	3.0 (2.0–4.0)	3.0 (2.0–4.0)	4.0 (3.0–5.8)	0.06

Data are shown as median (interquartile range (IQR)) for non-normally distributed continuous variables, while non-skewed variables are reported as mean ± standard deviation (SD). Abbreviations. BMI, body mass index; FSH, follicle-stimulating hormone; hpFSH: highly purified FSH; LH, luteinizing hormone; rhFSH: recombinant human FSH.

**Table 2:** Intra-group analysis of demographics, hormonal levels and testicular volume. Group 1 was treated with hpFSH followed by rhFSH, Group 2 with rhFSH followed by hpFSH, Group 3 with hpFSH followed by the same preparation, and Group 4 with rhFSH followed by the same preparation. The two different FSH preparations were administered at a dose of 150 IU three times a week for 3 months, and the second cycle of therapy was prescribed after a 3-month washout from the first cycle for all groups (as per the provisions of the Italian Pharmacopeia) (Supplementary Figure S1).

	Group 1 (n = 22)			Group 2 (n = 22)			Group 3 (n = 15)			Group 4 (n = 15)		
	T0	T1	T2	T0	T1	T2	T0	T1	T2	T0	T1	T2
BMI (kg/m^2^)	27.5 (23.0–34.0)	27.5 (23.0–34.0)	28.5 (23.0–34.0)	30.0 (20.0–33.0)	26.5 (23.0–34.0)	29.0 (23.0–31.0)	27.0 (23.3–33.8)	26.0 (22.5–33.5)	26.0 (22.0–33.5)	27.0 (23.0–33.8)	29.0 (22.0–33.5)	26.0 (22.0–33.5)
Testicular volume (mL)	11.1 ± 3.0	11.6 ± 2.6	13.4 ± 2.4 *^,†^	11.3 ± 2.5	11.9 ± 2.6	14.0 ± 1.9 *^,†^	11.0 (8.0–11.8)	11.0 (8.3–11.8)	11.0 (9.3–12.0)	9.6 ± 3.3	10.6 ± 2.0	12.0 ± 2.0 *
LH (mUI/mL)	2.8 ± 0.9	2.9 ± 0.8	3.3 ± 0.8	2.9 (2.0–3.0)	3.0 (2.8–3.4)	3.3 (3.0–4.0) *	3.1 ± 0.8	4.0 ± 1.4	5.0 ± 2.0 *	3.3 ± 1.0	4.5 ± 1.6 *	5.5 ± 1.8 *
FSH (mUI/mL)	3.1 ± 1.2	3.3 ± 1.0	4.1 ± 1.2 *^,†^	3.1 ± 1.1	3.4 ± 1.0	4.2 ± 1.4 *^,†^	2.8 (2.2–3.3)	3.3 (3.0–5.0)	5.0 (3.2–6.0) *	3.3 (2.4–4.2)	45.0 (3.3–6.0)	6.0 (3.5–6.0) *
Total testosterone (ng/dL)	482.5 (445.0–555.0)	473.0 (456.0–556.0)	566.5 (469.0–678.0) *^,†^	513.7 ± 79.4	548.1 ± 99.9	584.7 ± 114.7	522.4 ± 73.5	566.9 ± 103.3	589.5 ± 91.3	492.1 ± 80.0	596.5 ± 119.6 *	638.4 ± 124.3 *

Data are shown as median (interquartile range (IQR)) for non-normally distributed continuous variables, while non-skewed variables are shown as mean ± standard deviation (SD). * *p* < 0.05 vs. T0; ^†^ *p* < 0.05 vs. T1. Abbreviations. BMI, body mass index; FSH, follicle-stimulating hormone; hpFSH: highly purified FSH; LH, luteinizing hormone; rhFSH: recombinant human FSH.

**Table 3:** Inter-group analysis of the study parameters at time 1 (T1).

Parameters	Group 1	Group 2	Group 3	Group 4
Testicular volume (mL)	12.0 (10.0–14.0)	12.0 (10.0–14.0)	11.0 (8.3–11.8)	11.0 (9.3–12.0)
LH (mUI/mL)	2.9 ± 0.8	3.1 ± 0.8	4.0 ± 1.4 *^,†^	4.5 ± 1.6 *^,†^
FSH (mUI/mL)	3.2 (3.0–4.0)	3.4 (3.0–4.0)	3.3 (3.0–5.0)	5.0 (3.3–6.0) *
Total testosterone (ng/dL)	473.0 (456.0–556.0)	549.5 (456.0–613.0)	556.0 (467.5–675.0)	562.0 (479.8–694.5)
Sperm concentration (×10^6^/mL)	8.9 ± 2.5	9.4 ± 2.8	9.5 ± 2.7	8.7 ± 3.3
Total sperm count (×10^6^/ejaculate)	27.8 ± 10.0	27.8 ± 10.0	24.9 ± 7.8	29.9 ± 8.8
Sperm progressive motility (%)	11.0 (8.0–12.0)	11.0 (8.0–12.0)	11.0 (8.0–12.8)	11.0 (8.0–13.0)
Sperm morphology (%)	3.0 (2.0–3.0)	3.0 (2.0–3.0)	3.0 (3.0–4.8)	5.0 (2.3–6.5)

Data are shown as median (interquartile range (IQR)) for non-normally distributed continuous variable, while non-skewed variables are shown as mean ± standard deviation (SD). * *p* < 0.05 vs. Group 1; ^†^ *p* < 0.05 vs. Group 2. Abbreviations. FSH, follicle-stimulating hormone; hpFSH: highly purified FSH; LH, luteinizing hormone; rhFSH: recombinant human FSH.

## Data Availability

Data regarding any of the subjects in the study have not been previously published unless specified. Data will be made available to the editors of the journal for review or query upon request.

## Supplementary Materials

The following supporting information can be downloaded at: https://www.mdpi.com/article/10.3390/jcm13195666/s1, Supplementary Figure S1. Study protocol. Seventy-four infertile patients with abnormal sperm parameter and normal FSH levels were included in the study and were divided into 4 groups. Group 1 and Group 3 were treated with hpFSH for three months (150 IU three times a week), while Group 2 and Group 4 with rhFSH (150 IU three times a week). After the first cycle of treatment and a three-month washout (Group 1 and Group 2 were treated with a different FSH preparation, while Group 3 and Group 4 were treated with the same FSH preparation used for the first cycle for another three months (150 IU three times a week for both groups). Primary and secondary outcomes were assessed at baseline and at the end of the first and second cycle of treatment; Supplementary Table S1. Baseline parameters of the enrolled cohort consisting of infertile patients with abnormal sperm parameters and normal serum follicle-stimulating hormone (FSH) levels (<8 IU/mL); Supplementary Table S2. Inter-group analysis of the study parameters at time 2 (T2).
