# Supplementary material for: Effects of Switching FSH Preparations on Sperm Parameters and Pregnancy: A Prospective Controlled Study
Source: J Clin Med. 2024 Sep 24;13(19):5666. doi: 10.3390/jcm13195666 (PMC11477313; doi:10.3390/jcm13195666)
Supplement: Supplementary file 1 [file jcm-13-05666-s001.zip › jcm-3169309-supplementary.pdf]

Supplementary Tables

Supplementary Table 1. Baseline parameters of the enrolled cohort consisting of infertile patients with abnormal sperm parameters and normal serum follicle-stimulating hormone (FSH) levels (<8 IU/mL).

	n	Median	IQR	p-value*
Age (years)	74	33.0	26.0 to 37.0	0.0232
BMI (kg/m^2)	74	28.0	23.0 to 34.0	0.0003
Testicular volume (mL)	74	11.0	8.0 to 12.0	0.0012
LH (mUI/mL)	74	3.0	2.2 to 3.3	0.0018
FSH (mUI/mL)	74	3.0	2.2 to 4.0	0.0014
Total testosterone (ng/dL)	74	488.0	445.0 to 562.0	0.0015
Semen volume (mL)	74	2.0	2.0 to 3.0	<0.0001
Sperm concentration (mil/mL)	74	8.0	5.0 to 11.0	0.0003
Total sperm count (mil/ejaculate)	74	20.0	12.0 to 28.0	0.0114
Sperm progressive motility (%)	74	8.0	6.0 to 11.0	0.0047
Sperm morphology (%)	74	3.0	2.0 to 4.0	0.0002

Abbreviations. BMI, Body mass index; IQR, Interquartile range; LH, Luteinizing hormone. *Shapiro-Wilk test

Supplementary Table 2. Inter-group analysis of the study parameters at time 2 (T2). Group 1 was treated with hpFSH followed by rhFSH; Group 2 with rhFSH followed by hpFSH; Group 3 with hpFSH followed by the same preparation; and Group 4 with rhFSH followed by the same preparation. The two different FSH preparations were administered at a dose of 150 IU three times a week for 3 months and the second cycle of therapy was prescribed after a three-month washout from the first for all groups (as per the provisions of the Italian Pharmacopeia) (**Supplementary Figure 1**).

**Parameters**	**Group 1**	**Group 2**	**Group 3**	**Group 4**
Testicular volume (mL)	13.4±2.4	14.0±1.9	10.4±2.0*,†	12.0±2.0*,†
LH (mUI/mL)	3.3 (3.0-4.0)	3.3 (3.0-4.0)	5.0 (4.0-6.0)*	6.0 (4.0-6.0)*,†
FSH (mUI/mL)	4.0 (3.3-5.0)	3.9 (3.0-5.4)	5.0 (3.2-6.0)	6.0 (3.5-6.0)
Total testosterone (ng/dL)	590.4±111.8	584.7±114.7	589.5±91.3	638.4±124.3
Sperm concentration (mil/ml)	16.0±3.1	12.3±2.5*	10.6±2.1*	11.6±2.5*
Total sperm count (mil/ej.)	48.0 (44.0-60.0)	44.0 (30.0-48.0)	22.0 (20.0-32.8)*,†	36.0 (30.0-42.6)*
Progressive motility (%)	14.5 (11.0-25.0)	14.0 (11.0-22.0)	10.0 (8.0-12.5)*,†	12.0 (11.3-13.8)
Morphology (%)	2.455±1.224	2.955±1.090	2.933±1.438	3.867±1.642

Data were shown as median [interquartile range (IQR)] for non-normally distributed continuous variables; while non-skewed variables were as mean ± standard deviation (SD). The distribution of values was evaluated using the Shapiro-Wilk test. One-way analysis of variance (ANOVA) or the Kruskal-Wallis test were used for normally and non-normally distributed variables, respectively, to test the differences in continuous variables before and after FSH administration, with Dunn post hoc correction for multiple comparison tests. A p-value <0.05 was considered statistically significant.

*p<0.05 vs. Group 1; †p<0.05 vs. Group 2.

Abbreviations. FSH, follicle-stimulating hormone; hpFSH: Highly purified FSH; LH, luteinizing hormone; rhFSH: Recombinant human FSH.

Supplementary Figures

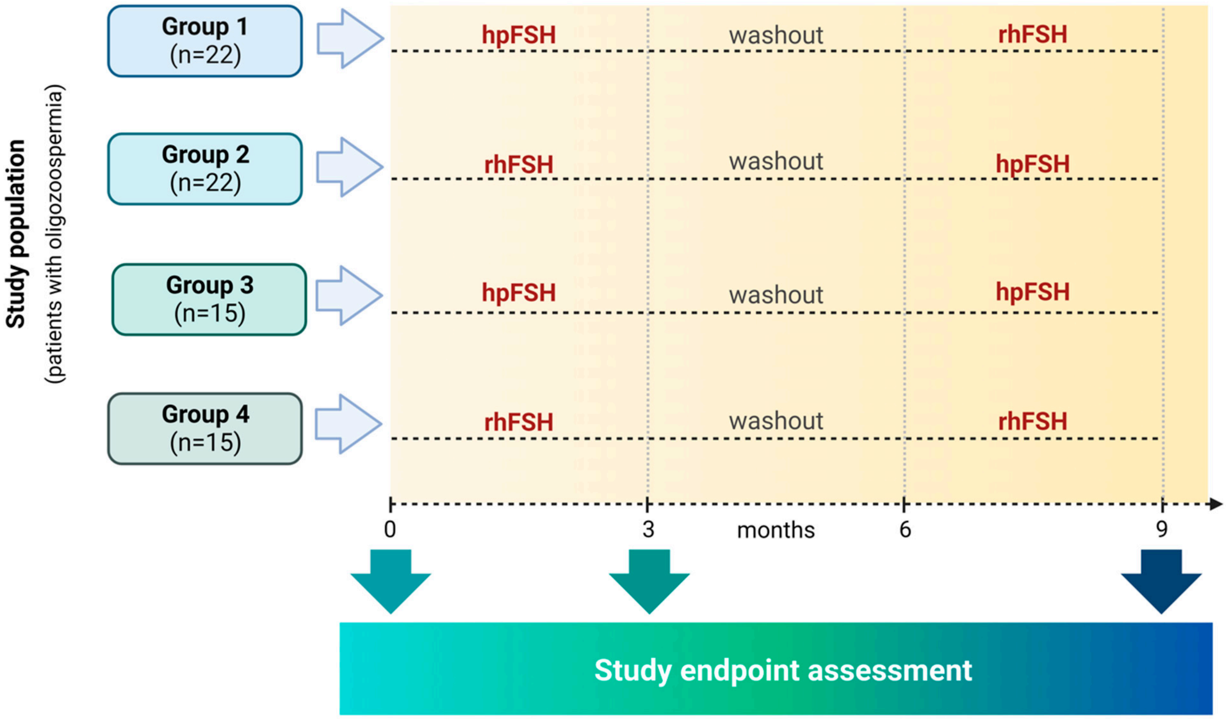

Supplementary Figure 1. Study protocol. Seventy-four infertile patients with abnormal sperm parameter and normal FSH levels were included in the study and were divided into 4 groups. Group 1 and Group 3 were treated with hpFSH for three months (150 IU three times a week), while Group 2 and Group 4 with rhFSH (150 IU three times a week). After the first cycle of treatment and a three-month washout (, Group 1 and Group 2 were treated with a different FSH preparation, while Group 3 and Group 4 were treated with the same FSH preparation used for the first cycle for another three months (150 IU three times a week for both groups). Primary and secondary outcomes were assessed at baseline and at the end of the first and second cycle of treatment. **Abbreviations.** FSH, Follicle-stimulating hormone; hpFSH: Highly purified FSH; rhFSH: Recombinant human FSH.
